# Association between γ-Glutamyl Transpeptidase and SARS-CoV-2 Spike Antibody Titers among BNT162b2 Vaccine Recipients

**DOI:** 10.3390/vaccines10122142

**Published:** 2022-12-14

**Authors:** Zobida Islam, Shohei Yamamoto, Tetsuya Mizoue, Yusuke Oshiro, Natsumi Inamura, Takeshi Nemoto, Maki Konishi, Mitsuru Ozeki, Wataru Sugiura, Norio Ohmagari

**Affiliations:** 1 Department of Epidemiology and Prevention, Center for Clinical Sciences, National Center for Global Health and Medicine, Toyama 1-21-1, Shinjuku-Ku, Tokyo 162-8655, Japan; 2 Department of Laboratory Testing, Center Hospital, National Center for Global Health and Medicine, Tokyo 162-8655, Japan; 3 Center for Clinical Sciences, National Center for Global Health and Medicine, Tokyo 162-8655, Japan; 4 Center Hospital, National Center for Global Health and Medicine, Tokyo 162-8655, Japan

**Keywords:** γ-glutamyl transpeptidase, COVID-19, SARS-CoV-2, vaccine, immunogenicity

## Abstract

Background: Increased γ-glutamyl transpeptidase (GGT) levels can deplete plasma glutathione, which in turn impairs immune regulation; however, evidence on GGT levels and post-vaccine immunogenicity is lacking. Objective: To examine the association between GGT and SARS-CoV-2 spike IgG antibodies. Methods: Participants were 1479 medical staff (aged 21 to 75 years) who received a SARS-CoV-2 antibody test after their second vaccine and whose GGT levels were measured before the vaccine rollout. Elevated and highly elevated GGT levels were defined as 51–80 and ≥81 U/L, respectively. Multivariable linear regression was used to calculate the means of SARS-CoV-2 spike IgG. Results: In a basic model, both elevated and highly elevated GGT levels were associated with significantly lower antibody titers. The ratio of mean (95% CI) was 0.83 (0.72–0.97) and 0.69 (0.57–0.84) for elevated and highly elevated GGT levels, respectively. However, these associations were largely attenuated after additional adjustment for potential confounders. An inverse association between GGT levels and antibody titers was found in women [0.70 (0.51–0.97)], normal-weight adults [0.71 (0.51–0.98)], and non-drinkers [0.73 (0.46–1.14)] but not in men, overweight adults, and alcohol drinkers. Conclusions: Circulating GGT concentrations were associated with the humoral immune response after COVID-19 vaccination, but this relationship could be ascribed to confounders such as sex, BMI, and alcohol drinking rather than GGT per se.

## 1. Introduction

Clinical studies showed that the COVID-19 vaccine is effective in preventing severe forms of COVID-19 [1]. Several studies examined the safety and immunogenicity of COVID-19 vaccines among patients with comorbidities (e.g., obesity, diabetes, and cardiovascular diseases) [2,3,4]. Since the liver has an important role in the adaptive/specific immune response through the direct or indirect priming of T and B lymphocytes and defending against harmful pathogens [5], liver dysfunction may interfere with vaccine-induced antibody production. However, the evidence on individuals with liver dysfunction is not sufficient

γ-Glutamyl transpeptidase (GGT), an enzyme localized in the biliary poles of hepatocytes and cholangiocytes [6], is a clinical marker of liver injury [7,8]. Moreover, GGT plays an important role in glutathione (GSH) metabolism by mitigating oxidative stress or inflammation and regulating innate immunity [9,10,11]. However, increased levels of GGT can deplete plasma GSH, which in turn impairs immune regulation [10,11]. Epidemiological studies showed that an increase in the risk of non-communicable diseases (e.g., diabetes, cardiovascular diseases, cancer, etc.) is associated with higher GGT levels [10,12]. Recent investigations also revealed a link between higher GGT levels and poor outcomes of COVID-19 [13,14,15]. 

In the post-vaccine era, whether elevated GGT levels interfere with vaccine-induced immunogenicity is of interest. To the best of our knowledge, only one study addressed this issue and reported significantly lower SARS-CoV-2 IgG antibody titers among those with higher GGT levels [16]. However, that study did not adjust for potentially important confounders, including age, gender, body mass index (BMI), and alcohol drinking. It has been reported that levels of GGT differ across age and gender [17,18] and are clinical markers of heavy alcohol drinking [19] and of abdominal fat distribution [20], all of which are known determinants of vaccine-induced immunogenicity [21,22,23]. Thus, it remains elusive whether the GGT-antibody association is confounded or modified by these variables. 

Here, we investigated the association between SARS-CoV-2 spike antibody titers after two doses of the BNT162b2 vaccine and pre-vaccinated GGT levels, considering potential confounders such as gender, alcohol drinking, and obesity. We additionally examined whether the association between GGT levels and antibody titers is modified by these factors.

## 2. Methods

### 2.1. Study Design

A repeat serological survey was launched among the staff of the National Center for Global Health and Medicine (NCGM), Japan, to monitor the spread of SARS-CoV-2 infection. The details of the study design are available elsewhere [24]. In brief, participants were asked to donate venous blood and answer a questionnaire on vaccination, history of COVID-19, health-related lifestyle, etc. Anti-SARS-CoV-2 nucleocapsid (from all surveys) and spike protein antibodies (from the second survey onward) were measured. Information on biochemical data and disease history were retrieved from annual health checkups conducted before the in-house vaccination program (Supplementary Figure S1). Written informed consent was obtained from each participant, and the study procedure was approved by the NCGM ethics committee.

### 2.2. Participants

For the present study, we used the data of the third survey (June 2021) conducted after the completion of the in-house vaccination program (COVID-19 mRNA-LNP BNT162b2; Pfizer-BioNTech) (Supplementary Figure S1). Of the 3072 workers invited to the survey, 2779 (90%) agreed to participate. Of these, 2474 participants had received two doses of the vaccine at least 15 days before the survey. Of these, 1563 participants had 2020 health checkup data for the study. We excluded 42 participants who had a history of COVID-19 (*n* = 8), tested positive with an anti-SARS-CoV-2 nucleocapsid protein assay (indicative of previous infection) (*n* = 12), or had a history of liver disease (*n* = 3) or cancer (*n* = 19). We then excluded those with missing data on GGT levels (*n* = 32), BMI (*n* = 9), or alcohol drinking (*n* = 1), leaving 1479 participants (aged 21–75 years) for the analysis.

### 2.3. Assessment of GGT

GGT levels were measured using the Japan Society of Clinical Chemistry (JSCC) standardization method (Kanto Chemical Co., Inc., Tokyo, Japan). We were not aware of any standard criteria for assessing GGT levels. Following guidelines of the Japan Society of Ningen Dock [25], we defined elevated (51–80 U/L) and highly elevated levels (≥81 U/L) of GGT. We also categorized the participants into GGT level quartiles.

### 2.4. Measurements of SARS-CoV-2 Spike Antibody Titers

IgG against the SARS-CoV-2 spike protein was detected by performing the AdviseDx SARS-CoV-2 IgG II assay using the Abbott ARCHITECT^®^, following the manufacturer’s instructions [24]. The assay detects the IgG antibodies against the receptor-binding domain (RBD) of the S1 subunit of the SARS-CoV-2 spike protein using a chemiluminescent microparticle immunoassay (CMIA). The resulting chemiluminescence, expressed in relative light units (RLU), indicates the strength of the response, which in turn reflects the quantity of IgG-S present. 

### 2.5. Assessment of Covariates

Since age [21], sex [21], BMI [22], smoking [26], alcohol consumption [23], and the number of days after the 2nd vaccination [24] are potential modifiers of vaccine-induced SARS-CoV-2 antibodies, we included those factors as covariates in our analysis. BMI was computed as weight in kilograms divided by height in meters squared. Daily alcohol consumption was estimated by the frequency (ranging from never to daily) and the amount consumed per day (ranging from <0.5 to ≥4 go/day). In Japan, *go* (180 mL) is used as the conventional unit to measure alcohol volume: 1 *go* of Japanese sake contains approximately 23 g of ethanol, which is equivalent to 500 mL of beer, 110 mL of shochu (25% alcohol content), a double (60 mL) of whisky, or 180 mL of wine. 

### 2.6. Statistical Analysis

Proportions and means were presented to show the background characteristics of the study population according to the status of GGT level. We transformed GGT levels and IgG spike antibody titers into a log scale before analysis. We employed a generalized linear regression model to estimate the ratio of mean and geometric mean IgG titers according to the status of GGT level. Model 1 was adjusted for the number of days after the 2nd vaccination (days, continuous). Model 2 was additionally adjusted for age (year, continuous) and sex (male or female). Model 3 was additionally adjusted for cigarette smoking (yes or no), BMI (kg/m^2^, continuous), and alcohol drinking (non-drinker, occasional drinker, <1 *go*/day, or ≥1 *go*/day). The estimates obtained were then back-transformed to present the geometric mean titer (95% CI) and the ratio of mean (95% CI). Additionally, we assessed antibody titers across GGT level quartiles. To examine whether the association between GGT levels and antibody titers differs depending on gender, alcohol drinking status, and being overweight (<23 kg/m^2^ or ≥23 kg/m^2^) according to the WHO classification of BMI for Asians [27], we performed a series of stratified analyses with these variables. Statistical significance was set at *p* < 0.05 for the trend. All analyses were performed using the statistical software Stata version 17.0 (StataCorp LLC, College Station, TX, USA).

## 3. Results

Table 1 shows participants’ characteristics according to GGT level status. Of participants, 5.2% and 3.3% had elevated and highly elevated GGT levels, respectively. Compared with those with normal levels of GGT, those with highly elevated levels were older, more likely to be male, smokers, and alcohol drinkers, and had a higher mean BMI, higher prevalence of dyslipidemia and diabetes, and higher median number of days after vaccination. 

As shown in (Table 2, Supplementary Figure S2), elevated and highly elevated GGT levels were significantly associated with decreased SARS-CoV-2 spike IgG antibody titers in a model adjusted for the duration after the second vaccination only (model 1). The geometric mean (95% CI) SARS-CoV-2 spike antibody titers was 5581 AU/mL (5386–5784), 4662 AU/mL (4016–5413), and 3870 AU/mL (3209–4666) for normal, elevated, and highly elevated GGT levels, respectively (*p* for trend < 0.001). The ratio of mean (95% CI) SARS-CoV-2 spike antibody titers was 0.83 (0.72–0.97) and 0.69 (0.57–0.84) for elevated and highly elevated GGT levels, respectively. However, these inverse associations were largely attenuated after additional adjustment for age and sex (model 2) and smoking, BMI, and alcohol drinking (model 3). The ratio of mean (95% CI) elevated and highly elevated GGT levels was 1.02 (0.88–1.19) and 0.89 (0.74–1.07), respectively, in model 2 and 1.06 (0.91–1.23) and 0.92 (0.76–1.10), respectively, in model 3. Similar results were found for GGT levels in the highest quartiles (Table 3, Supplementary Figure S2).

In the stratified analyses (Supplementary Table S1, Supplementary Figure S3), highly elevated GGT levels were associated with lower SARS-CoV-2 spike antibody titers in women but not in men. The ratio of mean (95% CI) highly elevated GGT levels was 0.70 (0.51–0.97) in women and 1.02 (0.81–1.29) in men. A significant inverse association between GGT levels and SARS-CoV-2 spike antibody titers was observed among normal-weight adults but not among overweight adults. The ratio of mean (95% CI) highly elevated GGT levels was 0.71 (0.51–0.98) among normal-weight adults and 1.01 (0.80–1.30) among overweight or obese adults. Antibody titers were decreased, albeit not statistically significantly, among those with highly elevated GGT levels in alcohol non-drinkers but not in alcohol drinkers.

## 4. Discussion

In the present study among hospital workers who completed two doses of the BNT162b2 vaccine, those with higher GGT levels had lower mean SARS-CoV-2 spike IgG antibody titers in the crude model. However, this association was largely attenuated after multivariable adjustment. In the stratified analyses, highly elevated GGT levels were associated with reduced SARS-CoV-2 spike IgG antibody titers in women, normal-weight adults, and non-drinkers but not in men, overweight or obese adults, and alcohol drinkers.

Our finding of an inverse association between GGT levels and antibody titers in a simple model is compatible with that of a Japanese study among vaccine recipients, which reported significantly higher median GGT levels in a group with lower antibody titers without adjustment for covariates [16]. However, the inverse association in our study was largely attenuated after adjustment for age, gender, BMI, and alcohol drinking, which are determinants of both GGT levels [17,18,19,20] and vaccine-induced immunogenicity [21,22,23]. This result suggests that the observed association observed in the simple model is largely driven by these confounding variables. 

In the stratified analyses, we found an inverse association between elevated GGT levels and antibody titers in the subgroups with lower mean GGT levels (women, alcohol non-drinkers, and normal-weight adults) but not in their counterparts. The reason for these findings is unclear. We speculate that higher circulating levels of GGT reflect lower levels of glutathione, which play an important role in immune response to viral infection [10,11], only among those free of these determinants. Additional studies are required to confirm whether GGT is a good marker of glutathione and thus associated with poor post-vaccine antibody productions in a group of people without known determinants of GGT. 

The strength of the present study includes its relatively large sample, adjustment for a wide range of potential covariates, and measurement of GGT levels prior to vaccine administration and antibody measurement, which precludes the possibility of reverse causation. This study also has some limitations that warrant mentioning. First, we did not measure neutralizing antibodies, which are reliable markers of the humoral immune response. Nonetheless, spike antibody titers measured with the assay we employed were well-correlated with the neutralizing antibody titers in vaccine recipients (Spearmen’s rank correlation coefficient was 0.91 and 0.83 for the wild-type and Delta live virus, respectively) [28]. Second, we did not measure cellular immune response, another key mechanism of disease control and infection protection [29]. Finally, study participants were apparently healthy, not primarily women (*n* = 16), alcohol non-drinkers (*n* = 8), and normal-weight adults (*n* = 14) in the group with highly elevated GGT levels, so caution should be exercised when generalizing these findings to other populations. 

In summary, the inverse association between circulating GGT levels and SARS-CoV-2 spike IgG antibody titers that was observed in a bivariable analysis was markedly attenuated after adjustment for age, gender, BMI, and alcohol drinking, suggesting that circulating GGT levels may not be independently associated with the humoral immune response after vaccination. The inverse association between GGT levels and antibody titers observed in the subgroups characterized by lower GGT levels (women, normal-weight adults, or alcohol non-drinkers) requires confirmation.

## Figures and Tables

**Table 1:** Participants’ characteristics according to γ-glutamyl transpeptidase level status.

	Γ-Glutamyl Transpeptidase		
	Normal	Elevated	Highly Elevated
Number of participants	1353	77	49
Age (mean ± SD, year)	32.8 ± 11.9	40.7 ± 11.4	43.2 ± 10.7
Men, *n* (%)	26.0	66.2	67.4
BMI (mean ± SD, kg/m^2^)	21.5 ± 3.2	24.5 ± 3.9	25.0 ± 3.0
Dyslipidemia, *n* (%)	22.3	46.7	73.5
Diabetes, *n* (%)	0.8	5.2	4.1
Current smoker, *n* (%)	6.7	13.0	8.2
Current alcohol drinker *, *n* (%)	36.7	68.8	73.5
Days after COVID-19 [median (IQR), days]	67 (55 to 70)	68 (43 to 70)	69 (62 to 70)

SD, standard deviation; IQR, interquartile range; BMI, body mass index. * More than 0 *go*/day alcohol drinking was defined as current alcohol drinking.

**Table 2:** Geometric mean (GMT) (95% CI) and ratio of mean (95% CI) SARS-CoV-2 spike antibody titers for each of the categories of γ-glutamyl transpeptidase level (according to the Japan Society of Ningen Dock, 2022).

	Number of Participants	SARS-CoV-2 Spike IgG Antibodies					
		GMT (95% CI)			Ratio of Mean (95% CI)		
		Model 1	Model 2	Model 3	Model 1	Model 2	Model 3
*γ-Glutamyl Transpeptidase*							
Normal	1353	5581 (5386–5784)	5477 (5295–5666)	5462 (5280–5651)	1.00 (Reference)	1.00 (Reference)	1.00 (Reference)
Elevated	77	4662 (4016–5413)	5609 (4852–6484)	5778 (4989–6690)	**0.83 (0.72–0.97)**	1.02 (0.88–1.19)	1.06 (0.91–1.23)
Highly elevated	49	3870 (3209–4666)	4870 (4062–5837)	5010 (4172–6017)	**0.69 (0.57–0.84)**	0.89 (0.74–1.07)	0.92 (0.76–1.10)
*p*^§^ for trend					**<0.001**	0.37	0.67

CI, confidence interval; GMT, geometric mean titer. Values in bold are statistically significant. Model 1 was adjusted for the number of days after the second vaccination (days, continuous). Model 2 was additionally adjusted for age (year, continuous) and sex (male or female). Model 3 was additionally adjusted for cigarette smoking (yes or no), BMI (kg/m^2^, continuous), and alcohol drinking (non-drinker, occasional drinker, <1 *go*/day, or ≥1 *go*/day). ^§^ Based on linear regression analysis, assigning ordinal numbers to the γ-glutamyl transpeptidase level categories.

**Table 3:** Geometric mean (GMT) (95% CI) and ratio of mean (95% CI) SARS-CoV-2 spike antibody titers for each of the γ-glutamyl transpeptidase level quartiles (according to the Japan Society of Ningen Dock, 2022).

	Number of Participants [Median (U/L)]	SARS-CoV-2 Spike IgG Antibodies					
		GMT (95% CI)			Ratio of Mean (95% CI)		
		Model 1	Model 2	Model 3	Model 1	Model 2	Model 3
Quartile 1	443 [13]	6363 (5983–6767)	5694 (5347–6063)	5618 (5273–5986)	1.00 (Reference)	1.00 (Reference)	1.00 (Reference)
Quartile 2	349 [16]	5630 (5253–6035)	5462 (5108–5840)	5405 (5053–5782)	**0.88 (0.81–0.97)**	0.96 (0.88–1.05)	0.96 (0.88–1.05)
Quartile 3	321 [22]	5237 (4872–5630)	5323 (4965–5707)	5365 (5004–5753)	**0.82 (0.75–0.91)**	0.93 (0.85–1.02)	0.95 (0.87–1.05)
Quartile 4	366 [39]	4579 (4279–4900)	5315 (4952–5705)	5419 (5037–5830)	**0.72 (0.66–0.79)**	0.93 (0.84–1.03)	0.96 (0.87–1.07)
*p*^§^ for trend					**<0.001**	0.14	0.44

CI, confidence interval; GMT, geometric mean titer. Values in bold are statistically significant. Model 1 was adjusted for the number of days after the second vaccination (days, continuous). Model 2 was additionally adjusted for age (year, continuous) and sex (male or female). Model 3 was additionally adjusted for cigarette smoking (yes or no), BMI (kg/m^2^, continuous), and alcohol drinking (non-drinker, occasional drinker, <1 *go*/day, or ≥1 *go*/day). ^§^ Based on linear regression analysis, assigning ordinal numbers to the γ-glutamyl transpeptidase level quartiles.

## Data Availability

All data supporting the findings of this study are available from the corresponding author upon reasonable request.

## Supplementary Materials

The following supporting information can be downloaded at: https://www.mdpi.com/article/10.3390/vaccines10122142/s1, Figure S1: Study design.; Figure S2: Geometric means of γ-glutamyl transpeptidase on the SARS-CoV-2 spike antibody titers.; Table S1: Ratio of mean (95% CI) of SARS-CoV-2 spike antibody titers according to the status of γ-glutamyl transpeptidase (according to the Japan Society of Ningen Dock. 2022) according to gender, drinking status, and BMI. Figure S3: Geometric means of γ-glutamyl transpeptidase on the SARS-CoV-2 spike antibody titers according to gender, alcohol drinking status, and body mass index (BMI) status.

## Abbreviations

γ-Glutamyl transpeptidase: GGT; body mass index: BMI; Japan Society of Clinical Chemistry: JSCC.
