# Supplementary material for: Association between γ-Glutamyl Transpeptidase and SARS-CoV-2 Spike Antibody Titers among BNT162b2 Vaccine Recipients
Source: Vaccines (Basel). 2022 Dec 14;10(12):2142. doi: 10.3390/vaccines10122142 (PMC9785427; doi:10.3390/vaccines10122142)
Supplement: Supplementary file 1 [file vaccines-10-02142-s001.zip › vaccines-2044124-supplementary.pdf]

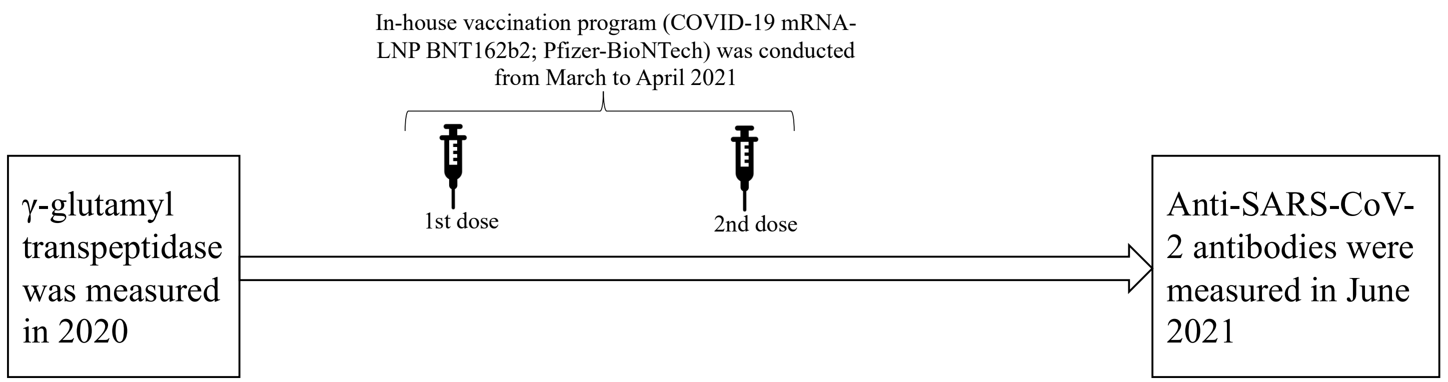

Supplementary Figure S1. Study design.

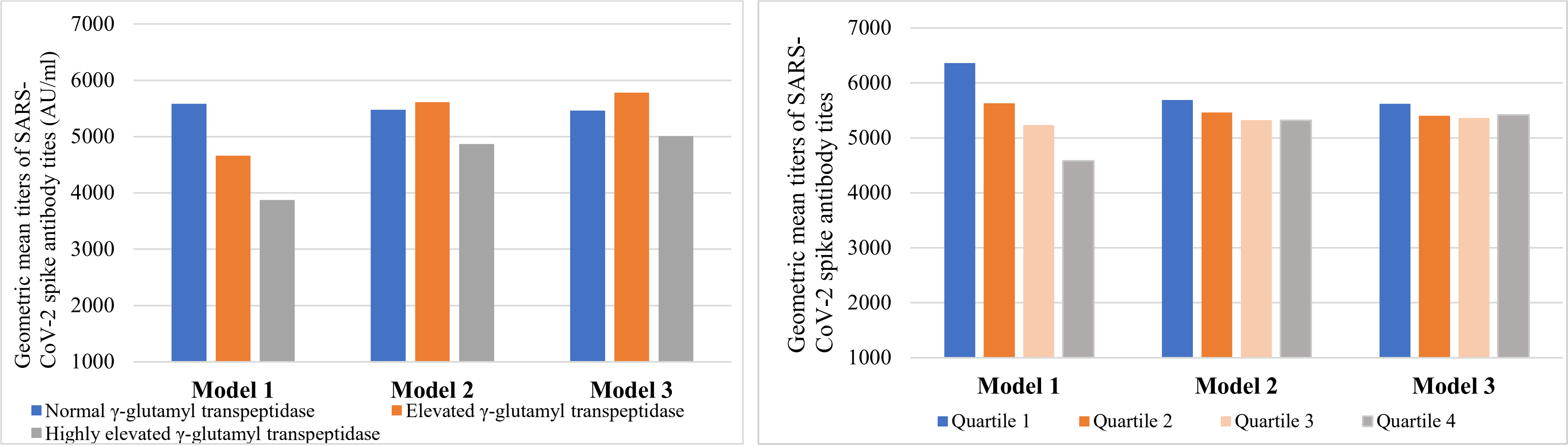

Supplementary Figure S2. Geometric means of γ-glutamyl transpeptidase on the SARS-CoV-2 spike antibody titers. Model 1 was adjusted for days after the second vaccination (days, continuous). Model 2 was additionally adjusted for age (year, continuous) and sex (male or female). Model 3 was additionally adjusted for cigarette smoking (yes or no), BMI (kg/m^2, continuous), and alcohol drinking (nondrinker, occasional drinker, <1 *go*/day, or ≥1 *go*/day). [§]Based on linear regression analysis, assigning ordinal numbers to the γ-glutamyl transpeptidase status.

Supplementary Table S1 Ratio of mean (95% CI) of SARS-Cov-2 spike antibody titers according to the status of γ -glutamyl transpeptidase (according to the Japan Society of Ningen Dock. 2022) according to gender, drinking status, and BMI

	Ratio of mean (95% CI) for SARS-CoV-2 spike IgG antibodies					
	Gender*		Alcohol drinking$		BMI$	
Number of participants	**Men**	**Women**	**Non-drinkers**	**Drinkers**	**<23kg/m²**	**≥23 kg/m²**
	436	1043	562	917	1056	423
Categories of-glutamyl transpeptidase						
Normal	1.00 (Reference)	1.00 (Reference)	1.00 (Reference)	1.00 (Reference)	1.00 (Reference)	1.00 (Reference)
Elevated	1.05 (0.87– 1.27)	1.02 (0.80– 1.31)	1.00 (0.75– 1.33)	1.03 (0.86– 1.24)	1.07 (0.85– 1.35)	1.03 (0.83– 1.27)
Highly elevated	1.02 (0.81– 1.29)	**0.70 (0.51– 0.97)**	0.73 (0.46– 1.14)	0.91 (0.73– 1.14)	**0.71 (0.51– 0.98)**	1.01 (0.80– 1.30)
P§ for trend	0.68	0.08	0.29	0.59	0.17	0.81
P for interaction		0.19		0.46		0.32
Quartiles of-glutamyl transpeptidase						
Quartile 1	1.00 (Reference)	1.00 (Reference)	1.00 (Reference)	1.00 (Reference)	1.00 (Reference)	1.00 (Reference)
Quartile 2	0.85 (0.65– 1.10)	0.97 (0.88– 1.07)	0.98 (0.85– 1.13)	0.95 (0.84– 1.07)	0.95 (0.86– 1.04)	1.01 (0.79– 1.29)
Quartile 3	0.90 (0.71– 1.15)	0.92 (0.83– 1.02)	0.97 (0.82– 1.14)	0.93 (0.83– 1.05)	**0.90 (0.81– 0.99)**	1.06 (0.84– 1.34)
Quartile 4	0.88 (0.70– 1.12)	0.93 (0.82– 1.06)	0.91 (0.77– 1.08)	0.96 (0.84– 1.09)	0.94 (0.83– 1.06)	1.01 (0.81– 1.26)
P§ trend	0.66	0.13	0.31	0.46	0.12	0.94
P for interaction		0.68		0.51		0.63

CI, confidence interval; *Model was adjusted for age (years, continuous) and days after the second vaccination (days, continuous). $Model was adjusted for age (year, continuous), sex (male or female), and days after the second vaccination (days, continuous). §Based on linear regression analysis, assigning ordinal numbers to the γ -glutamyl transpeptidase status.

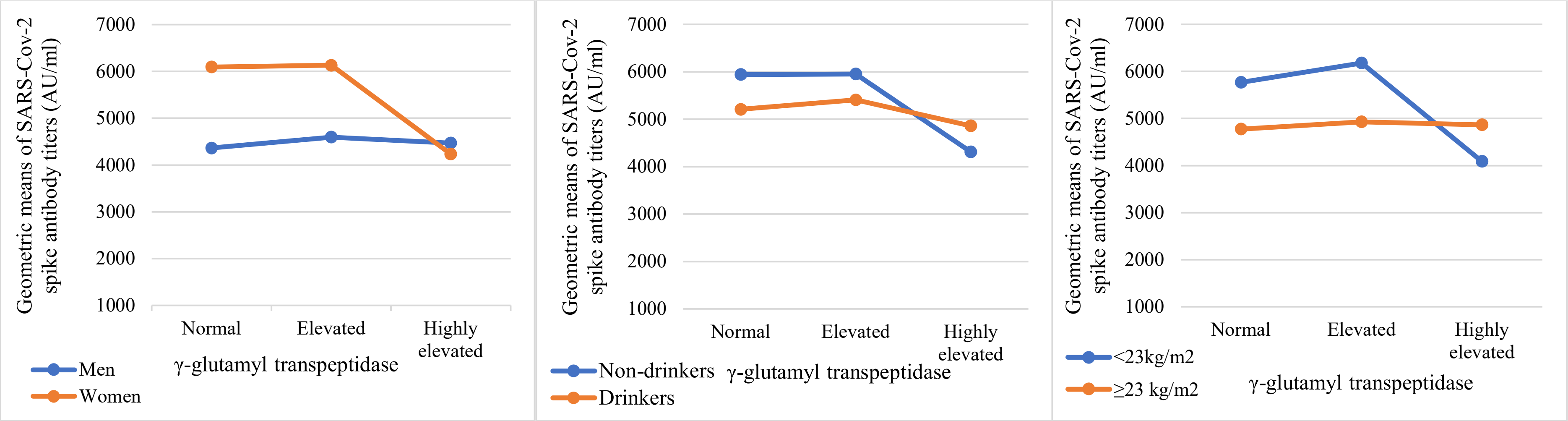

Supplementary Figure S3. Geometric means of γ -glutamyl transpeptidase on the SARS-CoV-2 spike antibody titers according to gender, alcohol drinking status, and body mass index (BMI) status. For the gender, the model was adjusted for age (year, continuous) and days after the second vaccination (days, continuous). For alcohol drinking status and BMI status, the model was adjusted for age (years, continuous), sex (male or female), and days after the second vaccination (days, continuous).
